# Identification of Changes in the Transcriptome Profile of Human Hepatoma HepG2 Cells Exposed to Combined Sorafenib and Cannabis Treatment

**DOI:** 10.3390/ijms27104342

**Published:** 2026-05-13

**Authors:** Krittakarn Udomkritayachai, Theeraphat Thiamsuk, Takdanai Jarujamrat, Panaphas Kudikhong, Sira Raksakhom, Phitsamai Suphattana, Natthanan Khankham, Palapoom Thanawong, Supakit Khacha-ananda

**Affiliations:** 1 Faculty of Medicine, Chiang Mai University, Chiang Mai 50200, Thailand; krittakarn_u@cmu.ac.th (K.U.); theeraphat_thi@cmu.ac.th (T.T.); takdanai_j@cmu.ac.th (T.J.); panaphas_kud@cmu.ac.th (P.K.); sira_raksakhom@cmu.ac.th (S.R.); 2 Department of Forensic Medicine, Faculty of Medicine, Chiang Mai University, Chiang Mai 50200, Thailand; phitsamai_su@cmu.ac.th (P.S.); natthanan2143@gmail.com (N.K.); 3 Multidisciplinary and Interdisciplinary School, Chiang Mai University, Chiang Mai 50200, Thailand; palapoom_th@cmu.ac.th

**Keywords:** cannabis, sorafenib, transcriptome, hepatocarcinoma cancer cells

## Abstract

Cannabis-derived compounds are increasingly used as adjuncts in cancer therapy due to their reported antiproliferative and pro-apoptotic effects. However, potential drug–herb interactions with standard anticancer agents—namely sorafenib—remain unclear. This study investigated the interaction between cannabis and sorafenib, together with transcriptomic alterations in human hepatoma HepG2 cells. Cell viability was assessed using the MTT assay, and drug interactions were evaluated using the Combenefit program. RNA sequencing was performed to characterize gene expression changes across treatment groups. Combination analysis demonstrated concentration-dependent synergistic effects at intermediate doses. Transcriptomic profiling revealed that the combination treatment induced a broader and more distinct set of differentially expressed genes compared with single treatments. Integrated enrichment analyses showed consistent activation of stress- and inflammation-related pathways, including tumor necrosis factor-α via nuclear factor-kappaB (TNF/NF-κB), mitogen-activated protein kinase (MAPK), janus kinase/signal transducers and activators of transcription (JAK–STAT), oxidative stress, and p53-mediated apoptosis, alongside suppression of metabolic and proliferative processes. While several pathways were shared across treatments, the combination group exhibited a more coordinated transcriptional response, including enrichment of integrated stress response, cytokine signaling, endoplasmic reticulum stress, and epigenetic regulation. These findings were supported by increased reactive oxygen species production and apoptosis, particularly in the combination group. Overall, cannabis may potentiate sorafenib activity through enhanced cellular stress and anti-proliferative signaling.

## 1. Introduction

Hepatocellular carcinoma (HCC) is a major global health burden and remains one of the leading causes of cancer-related mortality worldwide [1]. Sorafenib, a multi-kinase inhibitor, has been established as a first-line systemic therapy for advanced HCC; however, its clinical benefit is often limited due to the development of drug resistance [2,3]. These resistance mechanisms involve complex molecular alterations, including dysregulation of apoptotic signaling, metabolic adaptation, and activation of cytoprotective transcriptional programs. Such processes are closely associated with changes in gene expression, highlighting the importance of transcriptomic approaches to elucidate molecular responses to treatment and identify determinants of therapeutic efficacy [4].

Cannabis has gained increasing attention for its potential therapeutic applications in oncology, particularly following recent regulatory changes in several countries [5]. Its major bioactive compounds, Δ^9^-tetrahydrocannabinol (THC) and cannabidiol (CBD), have been reported to exert anti-inflammatory, antioxidant, and antineoplastic effects, including inhibition of cell proliferation, induction of apoptosis, and modulation of tumor-related signaling pathways [6,7]. Notably, emerging evidence indicates that cannabinoids interact with conventional chemotherapeutic agents, producing variable outcomes ranging from synergistic to antagonistic effects depending on drug combinations, concentrations, and cellular context [8,9]. In addition, variability in cannabis composition arising from differences in cannabinoid ratios could further influence these interactions and contribute to inconsistent biological responses [10]. Synergistic interactions enhance tumor cell sensitivity to chemotherapeutic agents by increasing oxidative stress, promoting apoptosis, and partially mitigating resistance mechanisms such as drug efflux and enhanced DNA repair capacity [11]. Conversely, antagonistic effects have been reported between cannabis and platinum-based drugs such as cisplatin by modulation of drug-metabolizing enzymes, attenuation of therapy-induced oxidative stress, activation of pro-survival signaling pathways and reduced induction of apoptosis [12,13].

Although previous reports demonstrate the use of cannabis in cancer patients under medical surveillance to alleviate treatment-related adverse effects, this growing co-use raises important concerns regarding potential drug–drug interactions with standard anticancer therapies [14]. Indeed, our findings underscore the need for caution when considering such combinatorial use, as the beneficial effects of cannabinoids in cancer therapy may not be universally applicable. In certain contexts, cannabis may attenuate the anticancer efficacy of chemotherapeutic agents, thereby potentially compromising treatment outcomes. This consideration is particularly important given the increasing prevalence of cancer patients using cannabis-derived products to alleviate treatment-related symptoms, highlighting the clinical relevance of understanding these interactions. However, despite the widespread clinical use of sorafenib in HCC and the increasing consumption of cannabis, their potential interaction remains poorly understood. Therefore, the present study aimed to address this knowledge gap by investigating the transcriptomic alterations associated with the combined treatment of cannabis and sorafenib in a human HCC cell line (HepG2). Using RNA sequencing (RNA-seq), we sought to characterize the molecular responses underlying this combination and to provide preliminary mechanistic insights into potential interactions between cannabis and sorafenib. While limited to an in vitro model, these findings may contribute to a better understanding of how cannabis could influence the response to sorafenib in HCC and inform future validation studies in more complex models.

## 2. Results

### 2.1. Composition of Cannabis Extract

The cannabinoid composition of the extract in our study showed that Δ^9^-tetrahydrocannabinol (Δ^9^-THC) accounted for 17.6256% (*w*/*w*), cannabinol (CBN) accounted for 1.6176% (*w*/*w*), and tetrahydrocannabinolic acid (THCA) accounted for 0.0280% (*w*/*w*). The total THC content, calculated as Δ^9^-THC + (THCA × 0.877), was 17.6502% (*w*/*w*). Cannabidiol (CBD) and cannabidiolic acid (CBDA) were present at 5.7393% (*w*/*w*) and 0.1429% (*w*/*w*), respectively, yielding a total CBD content, calculated as CBD + (CBDA × 0.877), of 5.8646% (*w*/*w*). Accordingly, the ratio of total THC to CBD was approximately 3:1.

### 2.2. Cannabis Suppresses Cancer Cell Proliferation

**Figure 1:**
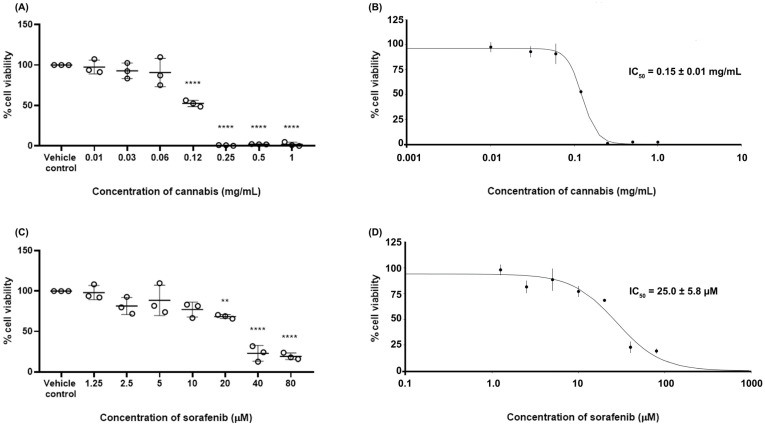
Cell viability of HepG2 cells following treatment with cannabis or sorafenib was assessed using the MTT assay after 24 h of incubation. (**A**,**C**) Percentage of cell viability is presented, and (**B**,**D**) sigmoidal dose–response curves were generated to determine IC_50_ values for each compound. Statistical significance is indicated as ** *p* ≤ 0.01 and **** *p* ≤ 0.0001 compared with the vehicle control (n = 3).

To determine the 50% inhibitory concentration (IC_50_) values of the test substances, the cell viability of HepG2 cells following treatment with cannabis extract or sorafenib was evaluated using the MTT assay. The results are shown in Figure 1. The cell viability in the vehicle control group was 100 ± 0.01%. Treatment with cannabis extract resulted in a concentration-dependent reduction in HepG2 cell viability, with statistically significant decreases observed at concentrations ranging from 0.12 to 1.0 mg/mL. Similarly, exposure to sorafenib led to a dose-dependent decrease in cell viability, with significant effects detected at concentrations between 20 and 80 µM. The determination of IC_50_ values for cannabis extract and sorafenib were approximately 0.15 ± 0.01 mg/mL and 25 ± 5.8 µM, respectively.

### 2.3. Synergistic Effect Between Cannabis and Sorafenib

**Figure 2:**
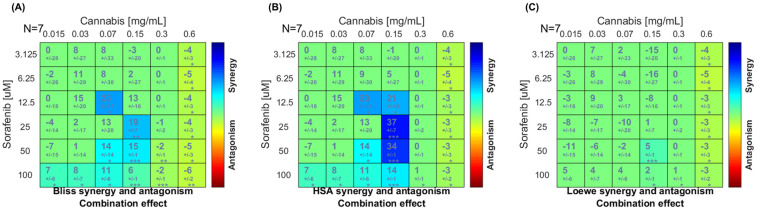
The drug–drug interaction between sorafenib and cannabis extract in HepG2 cells was analyzed using the Combenefit software. The plot showed synergy scores between sorafenib (*y*-axis) and cannabis extract (*x*-axis). Synergy surface maps were generated based on (**A**) the Bliss independence, (**B**) highest single agent (HSA), and (**C**) Loewe additivity models. Level of synergism or antagonism is indicated by color scale bars. Data was obtained from seven independent experiments (n = 7). * *p* < 0.05; ** *p* < 0.01, *** *p* < 0.001.

To characterize the pharmacological interaction between sorafenib and cannabis extract, a dose–matrix combination analysis was conducted using the Combenefit program. The cell viability in combination or single treatment is presented in Supplementary File S1. Figure 2 represents the synergy score in which color-coded synergy surface maps are used to visualize the degree of synergism or antagonism interactions between the two agents. As shown in the Bliss independence and highest single agent (HSA) synergy maps (Figure 2A,B), the strongest synergistic effects were observed at sorafenib concentrations of 12.5–25 μM in combination with 0.07–0.15 mg/mL of cannabis extract. In contrast, at the highest cannabis concentration tested (0.6 mg/mL), the interaction slightly shifted toward antagonism across most sorafenib concentrations. However, the Loewe additivity model (Figure 2C) indicated that the overall interaction between cannabis and sorafenib was largely additive across the dose–response matrix, with only synergistic effects detected at selected intermediate concentrations and several regions of mild antagonism, particularly at higher sorafenib doses. Finally, we conclude a concentration-dependent synergistic interaction between cannabis extract and sorafenib in HepG2 cells, with significant synergism at intermediate dose combinations. Based on the combination analysis, concentrations for RNA-sequencing were selected from the intermediate dose range, where the strongest and most consistent synergistic interactions were observed. Specifically, cannabis extract (0.07 and 0.15 mg/mL) in combination with sorafenib (12.5 μM) demonstrated high synergy scores across both Bliss and HSA models, while maintaining sufficient cell viability for downstream transcriptomic analysis.

### 2.4. Whole Gene Expression in Combined Sorafenib and Cannabis

To determine the changes in whole gene expression in HepG2 cells after treatment with a combination of sorafenib and cannabis extract, RNA-seq was performed. Venn diagram analyses were performed to compare the overlap and treatment-specific differentially expressed genes (DEGs) across control, single-agent, and combination treatment conditions. Across all comparisons, a large proportion of DEGs were commonly shared between all experimental groups.

**Figure 3:**
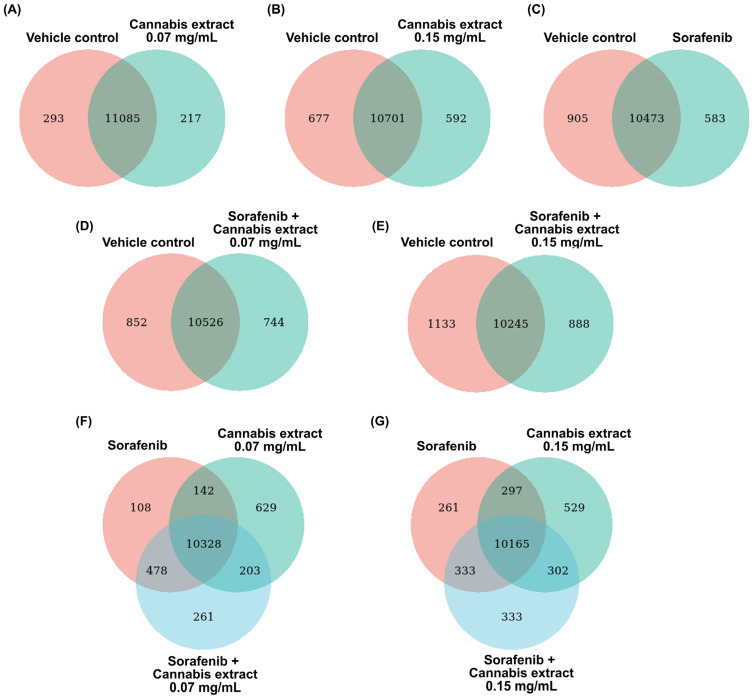
The Venn diagrams demonstrate the overlap and treatment-specific DEGs across control, single-agent, and combined treatment conditions relative to the vehicle control. (**A**) Cannabis extract (0.07 mg/mL) and vehicle control; (**B**) cannabis extract (0.15 mg/mL) and vehicle control; (**C**) sorafenib (12.5 μM) and vehicle control; (**D**) combination of sorafenib (12.5 μM) plus cannabis extract (0.07 mg/mL) and vehicle control; (**E**) combination of sorafenib (12.5 μM) plus cannabis extract (0.15 mg/mL) and vehicle control; (**F**) combination of sorafenib (12.5 μM) plus cannabis extract (0.07 mg/mL), sorafenib (12.5 μM), and cannabis extract (0.07 mg/mL); and (**G**) combination of sorafenib (12.5 μM) plus cannabis extract (0.15 mg/mL), sorafenib (12.5 μM), and cannabis extract (0.15 mg/mL) (n = 3).

In comparisons of the single treatments (cannabis extract or sorafenib alone) relative to the vehicle control (Figure 3A–C), distinct sets of DEGs were identified. Specifically, 217, 592, and 583 genes were uniquely associated with treatment using cannabis extract at concentrations of 0.07 and 0.15 mg/mL, and sorafenib, respectively. Furthermore, in comparisons of the combination treatments related to the vehicle control (Figure 3D,E), distinct DEGs sets were also observed. A total of 744 and 888 genes were uniquely associated with the combined treatment of cannabis extract (0.07 mg/mL) plus sorafenib, and cannabis extract (0.15 mg/mL) plus sorafenib, respectively. Of note, both combination treatments exhibited a greater number of unique DEGs compared with single-agent treatments, indicating enhanced transcriptomic modulation under combined drug exposure. Three-group Venn diagram analyses comparing sorafenib, cannabis extract (0.07 mg/mL), and the combination treatment (Figure 3F) demonstrated distinct DEG subsets, comprising 108 genes unique to sorafenib, 629 genes unique to cannabis extract, and 261 in the combination treatment. Finally, we also found 261 genes unique to sorafenib, 529 genes unique to cannabis extract (0.15 mg/mL), and 261 in the combination treatment (Figure 3G).

**Figure 4:**
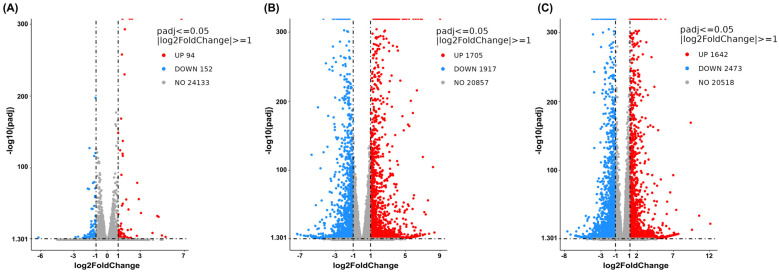
Volcano plots show global transcriptional changes following single treatment of cannabis extract or sorafenib relative to the vehicle control in HepG2 cells. The *x*-axis represents the log2-fold change in gene expression, and the *y*-axis indicates statistical significance expressed as −log10 adjusted *p*-value. (**A**) Cannabis extract (0.07 mg/mL), (**B**) cannabis extract (0.15 mg/mL), and (**C**) sorafenib (12.5 μM) (n = 3).

**Figure 5:**
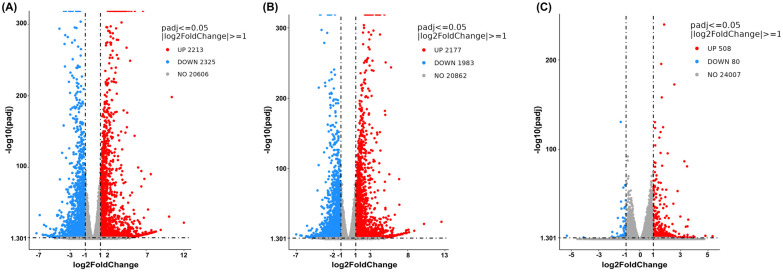
Volcano plots show global transcriptional changes following combined treatment of cannabis extract (0.07 mg/mL) and sorafenib (12.5 μM) in HepG2 cells. The *x*-axis represents the log2-fold change in gene expression, and the *y*-axis indicates statistical significance expressed as −log10 adjusted *p*-value. (**A**) Combined treatment with cannabis extract and sorafenib relative to vehicle control, (**B**) combined treatment with cannabis extract and sorafenib relative to cannabis extract alone, and (**C**) combined treatment with cannabis extract and sorafenib relative to sorafenib alone (n = 3).

**Figure 6:**
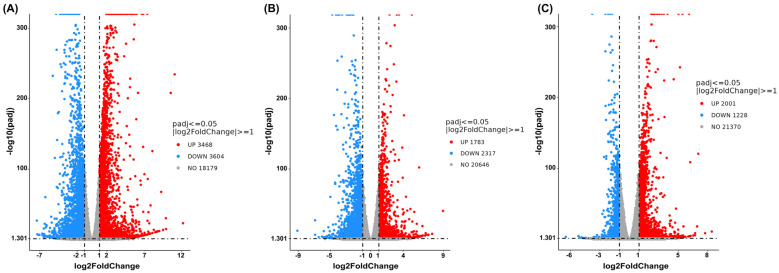
Volcano plots showing global transcriptional changes following combined treatment of cannabis extract (0.15 mg/mL) and sorafenib (12.5 μM) in HepG2 cells is shown. The *x*-axis represents the Log2-fold change in gene expression, and the *y*-axis indicates statistical significance expressed as −log10 adjusted *p*-value. (**A**) Combined treatment with cannabis extract and sorafenib relative to vehicle control, (**B**) combined treatment with cannabis extract and sorafenib relative to cannabis extract alone, and (**C**) combined treatment with cannabis extract and sorafenib relative to sorafenib alone (n = 3).

The DEG analysis was then visualized using volcano plots to demonstrate up- or downregulation changes between the treatment and vehicle control groups (Figure 4, Figure 5 and Figure 6). Comparisons between cells treated with cannabis extract at both tested concentrations and the vehicle control (Figure 4A,B) revealed concentration-dependent transcriptional deregulation, with both upregulated and downregulated genes. At the cannabis extract concentration of 0.07 mg/mL, the result showed 94 upregulated and 152 downregulated genes relative to the vehicle control (Figure 4A). Increasing the concentration up to 0.15 mg/mL of cannabis extract markedly expanded the transcriptional response, yielding 1705 upregulated and 1917 downregulated genes (Figure 4B). Also, a comparison between the cells treated with sorafenib and the vehicle control showed pronounced transcriptional alterations, with 1642 upregulated and 2473 downregulated genes (Figure 4C). Taken together, these results indicate that increasing concentrations of cannabis extract were associated with greater transcriptomic alterations.

We then made a comparison between the combination treatment (cannabis extract plus sorafenib) and the vehicle control or single treatment (single-cannabis extract or single-sorafenib treatment). At a concentration of 0.07 mg/mL of cannabis extract, the findings showed that the combined treatment resulted in 2213 upregulated and 2325 downregulated genes compared with the vehicle control (Figure 5A). We found that 2177 genes were upregulated and 1983 were downregulated in the combined treatment compared with single-cannabis extract at the same concentration (Figure 5B). On the contrary, we found fewer deregulated genes in the combined treatment compared with sorafenib monotherapy (508 upregulated and 80 downregulated genes) (Figure 5C). Overall, the transcriptional profile of the combination treatment showed limited additional changes in comparison with those observed with single treatments, particularly in comparison with sorafenib alone. Therefore, these findings suggest that cannabis extract at 0.07 mg/mL provides minimal additive or synergistic effects when combined with sorafenib. Accordingly, this concentration appears to have limited value for combination therapy and was not further investigated in subsequent experiments.

At a concentration of 0.15 mg/mL of cannabis extract, the result is shown in Figure 6, and a comparison between the combined treatment and the vehicle control revealed 3468 upregulated genes and 3604 downregulated genes (Figure 6A). Comparison of the combination treatment with the single-cannabis extract treatment identified 1783 upregulated genes and 2317 downregulated genes (Figure 6B). Finally, comparison between the combination treatment and the single-sorafenib treatment revealed 2001 upregulated genes and 1228 downregulated genes (Figure 6C). These results concluded that the combination treatment elicited a broader and more distinct transcriptional response than either the single-cannabis extract treatment or the single-sorafenib treatment alone. Hence, this combination formula was further chosen to analyze the molecular mechanisms.

**Figure 7:**
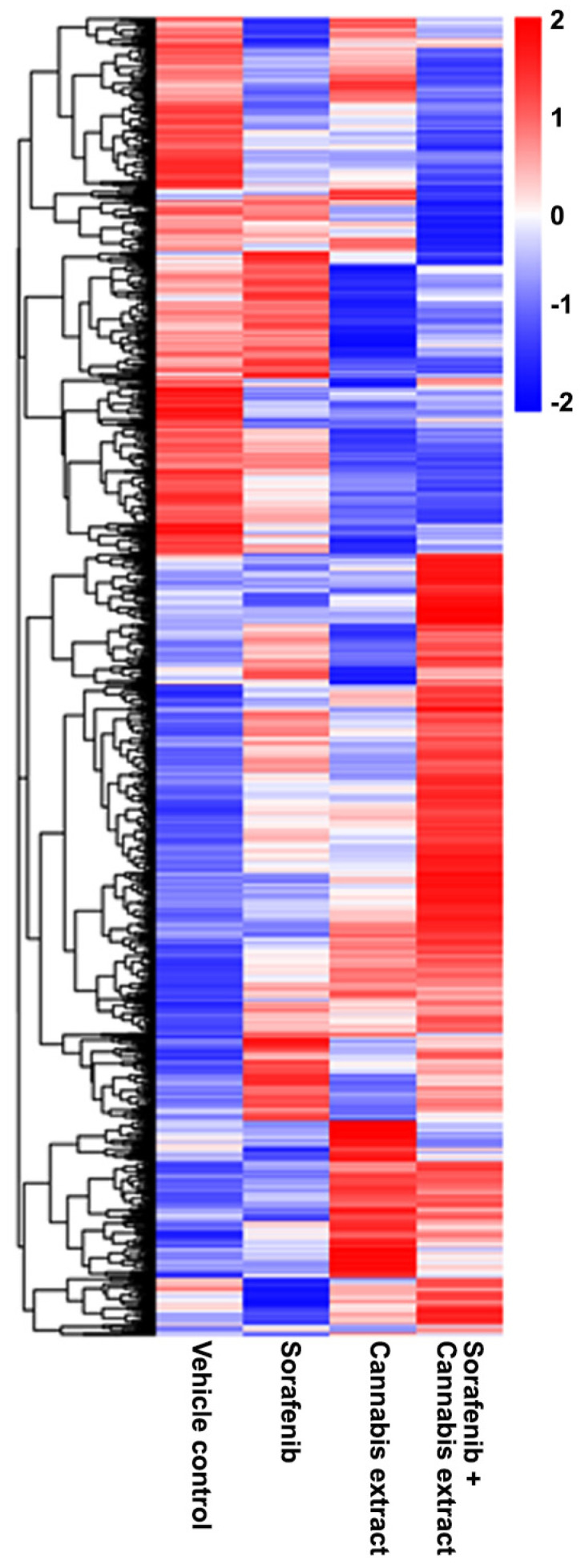
Differential expression gene clustering heatmap. The cells were treated with single-sorafenib extract, single-cannabis extract at a concentration of 0.15 mg/mL, or their combination. The overall results of FPKM cluster analysis, clustered using the log2(FPKM+1) value. Red indicates genes with high expression levels, and blue indicates genes with low expression levels (n = 3).

To explain the gene set exhibiting DEGs compared with the vehicle control, a heat map was generated to visualize the expression patterns of significantly DEGs across all experimental conditions, including the vehicle control and the cannabis extract (0.15 mg/mL), sorafenib (12.5 μM), and combined sorafenib–cannabis extract treatments (Figure 7). Each row represents an individual gene, and each column corresponds to a vehicle and treatment condition. Hierarchical clustering revealed that the cannabis extract group exhibited mixed gene expression patterns with an overall trend toward reduced expression. In contrast, the combined sorafenib–cannabis extract treatment formed a distinct cluster, showing gene expression changes that were opposite to those observed in the vehicle group. Moreover, the gene expression profile in the combined treatment differed from both single-agent treatments, indicating a unique transcriptional response. These findings suggest that cannabis extract modulates sorafenib-induced transcriptional programs rather than simply enhancing the effects of either agent alone.

The top 20 DEGs are presented in Table 1. Under single-treatment conditions, sorafenib induced the upregulation of several genes which were not detected in other groups, including *MT-RNR2*, *ACTB*, *NOTUM*, *ODC1*, *H3F3B*, *TRIB3*, *PIM1*, *SQSTM1*, *GNA13*, and *NFE2L2*. In contrast, treatment with cannabis extract alone resulted in the upregulation of *FTL*, *IGFBP1*, *TXNRD1*, *ERRFI1*, *DDX21*, *GCLC*, *HSPA9*, *H1F0*, *SERPINE1*, and *ATF4*. Notably, the combination treatment induced the upregulation of several genes that were not detected in either single-treatment condition, including *MT-RNR1*, *SAT1*, *BRD2*, and *MTHFD2.* Regardless, sorafenib-treated cells resulted in the downregulation of multiple genes, including *SLC2A1*, *HSPA8*, *LDHA*, *IGFBP1*, *LYZ*, *SLC2A3*, *MAN1A1*, *PEG3*, *HNRNPA2B1*, *PGK1*, *RPS6KA3*, *SLC16A3*, *PGAM1*, *PLIN2*, *NORAD*, *TM4SF1*, and *IDH1*. On the contrary, cannabis-treated cells showed downregulation of *AFP*, *TF*, *PEG10*, *FGG*, *TPI1*, *FGA*, *APOH*, *FGB*, *P4HA1*, *HMGA2*, *SERPINF2*, *PLOD2*, *ANXA4*, *TNFRSF19*, *COL2A1*, and *OBSL1*. Furthermore, several genes were uniquely downregulated in the combination treatment and were not detected in either single-treatment group, including *SLC2A1*, *SELENOP*, *AGT*, *SERPIND1*, *CPLX2*, *MMD*, *MCM3*, *PLOD2*, and *ENC1*.

**Figure 8:**
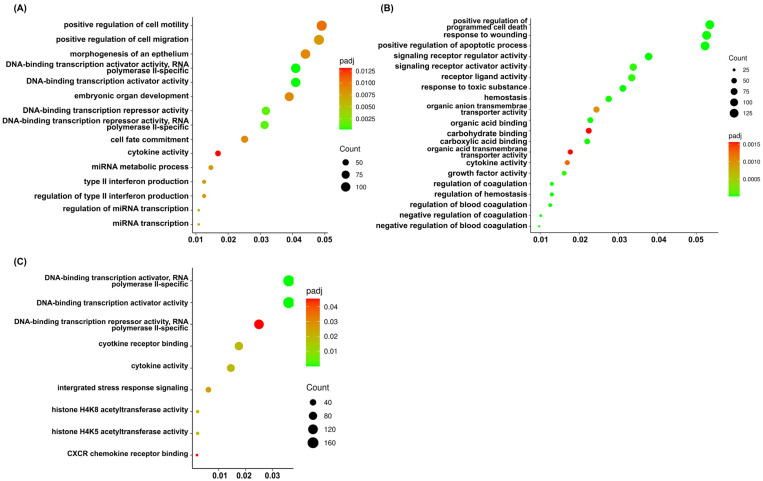
GO terms among treatment groups were analyzed. DEGs from each treatment group were subjected to GO enrichment analysis by clusterProfiler (4.8.1). GO terms that were uniquely enriched or shared among the three treatment conditions, based on FDR-adjusted significance, are presented. The *x*-axis represents gene ratio. (**A**) sorafenib treatment alone, (**B**) cannabis extract treatment alone, and (**C**) combination treatment with sorafenib and cannabis extract. The figure was generated using ImageGP (https://www.bic.ac.cn/ImageGP/, accessed on 3 April 2026) (n = 3).

To further characterize the biological functions associated with the DEGs, Gene Ontology (GO) enrichment analysis was performed. The significant genes identified from each treatment group were mapped to the database for identification of enriched functional categories, including biological processes (BPs), molecular function (MF), and cellular component (CC). The results are shown in Figure 8 and Supplementary File S2. Regarding the sorafenib treatment, we found a significant association with key regulators of BPs, particularly miRNA response, immune response, and cellular dynamics. In the MF category, genes were predominantly enriched in DNA-binding transcription regulatory activities and cytokine activity. The cannabis-treated cells showed that the BPs correlated with a strong overrepresentation of genes involved in responses to toxic substances and positive regulation of the apoptotic process as well as programmed cell death. Moreover, we found enriched terms related to hemostasis and blood coagulation. The MF category demonstrated genes associated with signaling receptor regulator/activator activity and receptor ligand activity as well as growth factor activity and cytokine activity. Additionally, enrichment in organic acid binding and transmembrane transporter activity was observed.

Interestingly, the cells were incubated with a combination of sorafenib and cannabis extract showed that gene sets were significantly related to the BPs in integrated stress response signaling and mapping to MF categories revealed strong enrichment in DNA-binding transcription activator and repressor activities and cytokine and chemokine activity as well as histone acetyltransferase. Remarkedly, GO terms including integrated stress response signaling, cytokine receptor binding, histone H4K5 acetyltransferase activity, histone H4K8 acetyltransferase activity, and CXCR chemokine receptor binding were predominantly enriched exclusively in this combination group and were not observed in either the sorafenib or cannabis extract treatment alone. These findings indicate the involvement of stress adaptation, epigenetic regulation, inflammatory signaling, and chemokine-mediated interactions, which are key processes underlying cancer cell survival, proliferation, immune evasion, and metastasis.

**Figure 9:**
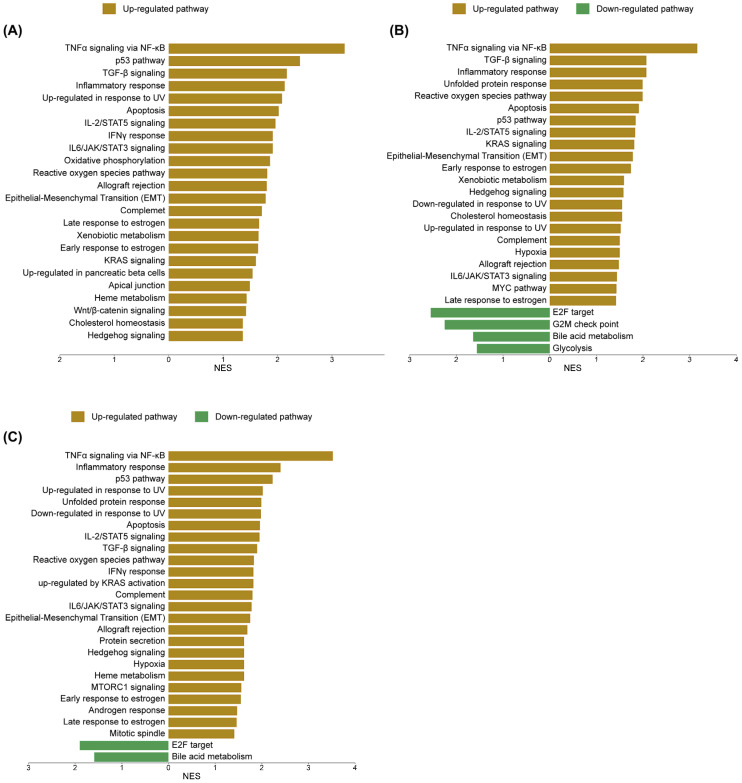
Gene Set Enrichment Analysis (GSEA) of differentially expressed genes from (**A**) sorafenib treatment alone, (**B**) cannabis extract treatment alone, and (**C**) combination treatment with sorafenib and cannabis extract. Enrichment analysis was performed using the h.all.v5.1.symbols.gmt [Hallmark] gene set collection with 1000 permutations. Gene sets with nominal *p*-value < 0.05 and FDR q-value < 0.05 were considered significantly enriched. Enrichment plots were generated using SR plot (http://www.bioinformatics.com.cn/Srplot, accessed on 6 May 2026) (n = 3).

To elucidate the molecular mechanisms underlying sorafenib and cannabis treatment in HepG2 cells, transcriptomic alterations were systematically analyzed using gene set enrichment approaches based on Gene Set Enrichment Analysis (GSEA) and ShinyGO 8.0. Regarding the GSEA database, the sorafenib treatment resulted in a marked transcriptional shift. The most prominently enriched pathways included TNFα signaling via NF-κB, p53 signaling, transforming growth factor-beta (TGF-β) signaling, inflammatory response, and ultraviolet (UV) response, indicating activation of stress and apoptosis-related processes. The cannabis treatment induced a comparable yet distinct transcriptional profile, with 34 of 50 gene sets upregulated and 16 downregulated. While overlapping with sorafenib in key pathways such as TNFα signaling via NF-κB, TGF-β signaling, and inflammatory response, cannabis uniquely enhanced unfolded protein response and reactive oxygen species pathways, highlighting its role in promoting toxicity to protein and oxidative stress. Consistently, the downregulated pathways included the G2/M checkpoint, further supporting the disruption of cell cycle control. Notably, the combination treatment elicited a more integrated and distinct transcriptional reprogramming, with 39 of 50 gene sets upregulated and 11 downregulated. This profile was characterized by a robust activation of the p53 pathway in conjunction with TNFα signaling via NF-κB, inflammatory response, UV response, and unfolded protein response, suggesting a convergence of stress, inflammatory, and apoptotic signaling. Meanwhile, persistent downregulation of E2F targets and bile acid metabolism across all treatment conditions indicates a shared suppression of proliferative and metabolic pathways. The upregulated and downregulated pathways are shown in Figure 9. The enrichment score (ES), normalized enrichment score (NES) and false discovery rate q-value (FDR q-value) are shown in Supplementary File S3.

**Figure 10:**
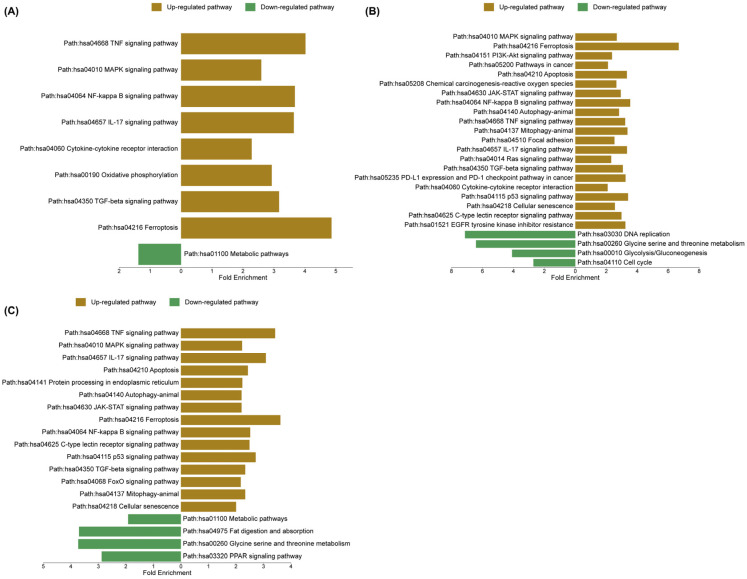
Top significantly enriched Kyoto Encyclopedia of Genes and Genomes (KEGG) pathways based on differentially expressed genes (DEGs) in HepG2 cells compared with the vehicle control group. (**A**) Sorafenib treatment alone, (**B**) cannabis extract treatment alone, and (**C**) combination treatment with sorafenib and cannabis extract. KEGG pathway enrichment analysis was performed using ShinyGO (v0.85.1), and enrichment plots were visualized using SR plot (http://www.bioinformatics.com.cn/Srplot, accessed on 6 May 2026) (n = 3).

To validate and extend the GSEA findings, ShinyGO 8.0 was utilized to compare pathway enrichment profiles and to determine whether the transcriptional responses across treatments reflect common regulatory mechanisms or distinct biological processes. In the sorafenib-treated group, upregulated pathways were strongly enriched in inflammatory and stress-related signaling, including TNF signaling, MAPK signaling, NF-κB signaling, interleukin-17 (IL-17) signaling, and cytokine–cytokine receptor interaction. In contrast, metabolic pathways were significantly downregulated, indicating a global suppression of cellular metabolic activity. In the cannabis-treated group, enriched pathways included MAPK signaling, ferroptosis, phosphoinositide 3-kinase/protein kinase B (PI3K–Akt) signaling, pathways in cancer, and apoptosis, suggesting activation of both stress response and cell death mechanisms. Meanwhile, downregulated pathways comprised DNA replication, glycine/serine/threonine metabolism, glycolysis/gluconeogenesis, and cell cycle progression, reflecting an inhibition of proliferative and metabolic functions. Notably, the combination treatment exhibited a convergent yet distinct enrichment profile, characterized by upregulation of TNF signaling, MAPK signaling, IL-17 signaling, apoptosis, and protein processing in the endoplasmic reticulum, indicating an integration of inflammatory, apoptotic, and endoplasmic reticulum stress responses. Concurrently, downregulated pathways included metabolic pathways, fat digestion and absorption, amino acid metabolism, and peroxisome proliferator-activated receptor (PPAR) signaling, further supporting the suppression of metabolic and lipid regulatory processes. The upregulated and downregulated pathways are shown in Figure 10. The enrichment FDR, number of genes, and fold enrichment are shown in Supplementary File S4.

**Figure 11:**
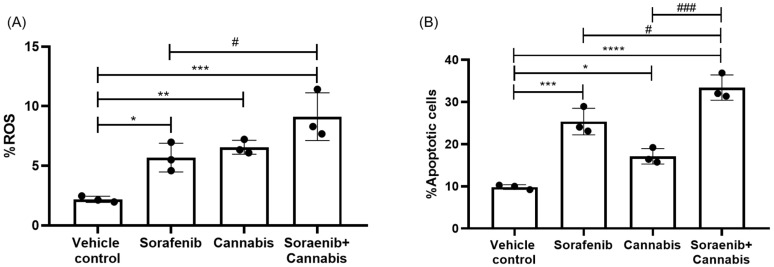
Functional assays assessing (**A**) reactive oxygen species (ROS) generation and (**B**) apoptosis in HepG2 cells following treatment with sorafenib alone, cannabis alone, or their combination. Fluorescently stained cells were quantified by flow cytometry and expressed as percentages. Data are presented as mean ± SD (n = 3). Statistical significance is indicated as * *p* ≤ 0.05, ** *p* ≤ 0.01, *** *p* ≤ 0.001, and **** *p* ≤ 0.0001 versus vehicle control, and # *p* ≤ 0.05 and ### *p* ≤ 0.001 versus the corresponding single-treatment groups.

Following RNA-sequencing analysis, we performed functional assays focusing on reactive oxygen species (ROS) production and apoptosis as key downstream phenotypic outcomes in cancer cells under stress induction. All treatment conditions resulted in a significant increase in the percentage of ROS-positive cells compared with the vehicle control group. Notably, the combination treatment further significantly elevated ROS levels compared with sorafenib monotherapy (Figure 11A). Similarly, apoptosis analysis revealed a significant increase in the proportion of apoptotic cells in response to single-agent treatment with either cannabis or sorafenib, as well as their combination, relative to the vehicle control. Furthermore, the combination treatment significantly enhanced apoptosis compared with either sorafenib or cannabis alone (Figure 11B).

## 3. Discussion

Cannabis has been widely reported to provide therapeutic benefits in the management of symptoms such as pain, inflammation, anorexia, mood disorders, and vomiting. Recently, increasing attention has been directed toward its potential role in cancer therapy, particularly due to its ability to alleviate chemotherapy-related side effects and possibly enhance treatment efficacy in resistant cases [15]. Nevertheless, concerns regarding potential drug–drug interactions should be carefully evaluated to determine whether cannabis affects the pharmacological properties of conventional anticancer drugs [16]. Although previous studies have demonstrated the effects of cannabis on the efficacy of several chemotherapy drugs, such as cisplatin, tamoxifen, doxorubicin, and paclitaxel, the molecular effects of combined administration of sorafenib and cannabis have not yet been reported. This study is the first to comprehensively investigate the drug–drug interaction between cannabis and sorafenib, and to characterize the transcriptomic effects of this combination in HepG2 cells, revealing associated biological responses and molecular pathways.

The cytotoxic analysis demonstrated that cannabis extract exhibited an IC_50_ value approximately 0.15 mg/mL after 24 h of exposure, corresponding to THC and CBD concentrations of 84.1 µM and 7.72 µM, respectively. When compared to other studies, we found that the recent IC_50_ is higher than that reported in previous studies. It has been suggested that differences in extract composition, particularly the relative proportions of THC and CBD as well as potential variations in cancer types, could reflect distinct effects on cancer treatment [16]. The different mechanisms of action by CBD and THC depend on the ratio between CBD and THC in cannabinoids [17]. Co-administration of tamoxifen with both recreational cannabis (THC:CBD ratio of 9:1) and medicinal cannabis (THC:CBD ratio of 1:3) formulations led to a reduction in tamoxifen’s efficacy, indicating that the simultaneous treatment of a breast cancer cell line with different cannabinoid formulations and tamoxifen results in a diminished anti-proliferative activity of tamoxifen [18]. Moreover, the pro-tumor effect of cannabis was related to the expression of cannabinoid receptors (CB), both CB1 and CB2; then, the cancer cells which originated from tissues with low cannabinoid receptor expression could be less sensitive to cannabinoids [19,20]. Blasco-Benito et al., 2018 [21], reported that cannabis exhibited dual effects depending on the anticancer agent. No synergy was observed with paclitaxel or epirubicin, whereas cisplatin combined with cannabinoid extract showed a synergistic inhibition of cancer cell growth.

The potential interaction between cannabis extract and sorafenib in HepG2 cells was evaluated using the Combenefit platform. The combination treatment at IC_50_ concentrations demonstrated a synergistic reduction in cell viability, as indicated by both the Highest single agent (HSA) and Bliss independence models. The HSA model provides a conservative baseline comparison without assuming mechanistic interaction [22], whereas the Bliss model predicts additive effects for compounds acting through independent mechanisms [23,24]. Hence, these findings indicate that the interaction between sorafenib and cannabis was predominantly synergistic, likely driven by distinct yet complementary mechanisms, with partial overlap in their modes of action. Mechanistically, sorafenib functions as a multikinase inhibitor targeting receptor tyrosine kinases and downstream serine/threonine kinases, resulting in inhibition of cell proliferation and angiogenesis, as well as induction of apoptosis [25]. In contrast, cannabinoids exerted anticancer effects through activation of CB1 and CB2 receptors, leading to suppression of cell growth, induction of cell cycle arrest, and activation of apoptotic pathways via multiple signaling cascades [26]. Previous studies reported synergistic anti-tumor effects when cannabinoids were combined with tyrosine kinase inhibitors, such as the synergistic effect of CBD and dasatinib through modulation of the PI3K-AKT pathway [27] as well as inhibition of human epidermal growth factor receptor (HER2) signaling [28]. Taken together, our findings suggest that cannabis extract enhanced sorafenib efficacy through both complementary and partially overlapping mechanisms.

At the mechanistic level, the combined effects of sorafenib and cannabinoids appeared to join in the induction of cellular stress. These include oxidative stress, mitochondrial dysfunction, and disruption of cellular homeostasis, ultimately leading to apoptosis and ferroptosis [29,30]. Cannabinoid-derived compounds such as β-caryophyllene showed the potential enhancement of sorafenib cytotoxicity by inhibiting drug efflux and increasing intracellular drug accumulation [31]. Combination treatments with cannabinoids and cisplatin have also been associated with suppression of DNA replication and repair, as well as induction of cell cycle arrest [32]. Notably, synergistic interactions with tyrosine kinase inhibitors, including cabozantinib, have been linked to activation of p53 signaling and endoplasmic reticulum (ER) stress, further promoting apoptotic cell death [33]. Likewise, chemotherapy in the class of platinum-based drugs exerted the induction of ROS in the mitochondria of gastric cancer cells leading to loss of mitochondrial membrane potential. As a consequence, the accumulation of misfolded proteins triggered the unfold protein response in an attempt to maintain cellular homeostasis [34]. Given that both sorafenib and cannabinoids are metabolized by cytochrome P450, family 3, subfamily A (CYP3A) enzymes, pharmacokinetic interactions may additionally contribute to the observed effects [35,36]. These findings support a multifaceted mechanism underlying the interaction, while highlighting the importance of transcriptomic analysis to further elucidate system-level responses.

RNA-sequencing analysis revealed that the combination treatment induced a distinct transcriptional profile, characterized by upregulation of stress- and apoptosis-related genes, for example, *SAT1* and *MTHFD2*, and downregulation of genes involved in proliferation, metabolism, and tumor progression such as *SLC2A1*, *MCM3*, *PLOD2*, and *ENC1*. These gene expression changes are consistent with suppression of metabolic reprogramming, inhibition of cell proliferation and angiogenesis, and activation of cell death pathways. For instance, upregulation of *SAT1* affected the reduction in polyamine levels resulting in the inhibition of tumor growth through AKT/GSK3β/β-catenin signaling [37,38], while downregulation of *AGT*, *MCM3*, *PLOD2*, and *ENC1* has been reported to associate with decreased tumor proliferation, invasion, and epithelial–mesenchymal transition [39,40,41,42,43]. Importantly, these gene-level alterations are highly consistent with pathway-level enrichment identified by GSEA and ShinyGO, collectively indicating coordinated suppression of metabolic programs and activation of stress- and apoptosis-related signaling. Consistent with these gene-level alterations, GO enrichment analysis further demonstrated that the combination treatment prominently activates biological processes related to transcriptional regulation and stress adaptation. Notably, enrichment of integrated stress response (ISR) signaling, cytokine and chemokine receptor binding, and histone acetyltransferase activity was uniquely observed in the combination group. The ISR represents a key adaptive mechanism to cellular stress such as hypoxia, nutrient deprivation, and endoplasmic reticulum stress resulting in shifting from pro-survival to pro-apoptotic signaling under sustained stress conditions [44,45]. In parallel, cytokine and chemokine signaling pathways reflect dynamic regulation of tumor–microenvironment interactions, influencing processes such as inflammation, immune response, angiogenesis, and metastasis [46]. Importantly, enrichment of histone acetyltransferase activity which serves as key processes in DNA repair, apoptosis, and tumor progression highlights a role for epigenetic regulation in mediating the observed transcriptional reprogramming [47,48]. Consistent with previous studies, both cannabinoids and sorafenib were reported to modulate epigenetic landscapes associated with inflammation and stress responses [49,50,51]. Hence, the integration of gene expression and GO enrichment findings suggests that the combination treatment induced a coordinated anticancer response involving metabolic suppression, activation of stress and immune-related signaling, and epigenetic reprogramming. These multi-layered mechanisms likely contribute to the enhanced anti-proliferative and pro-apoptotic effects observed in HepG2 cells.

Integrated analysis of GSEA and the ShinyGO database showed that pathway enrichment analysis revealed that key signaling networks, including TNF/NF-κB, MAPK, JAK/STAT, ROS, and p53 pathways, were activated across the treatments, with stronger and more coordinated activation observed in the combination group. This pattern suggests that the combination amplifies shared stress and apoptotic signaling rather than inducing entirely distinct pathways. In the sorafenib-treated cells, we found significantly upregulated stress- and inflammation-associated pathways (TNF/NF-κB, MAPK, JAK/STAT, and TGF-β signaling). Despite activation of these signaling pathways related to tumor progression and chemoresistance, it likely reflected compensatory and adaptive responses rather than pro-tumorigenic effects under therapeutic stress. At the cellular level, some cells might be induced to undergo apoptosis, while other cells might activate NF-κB/STAT3/PI3K-driven resistance programs to promote survival [52]. In addition, the simultaneous activation of stress-related pathways and suppression of metabolic processes by the ShineyGo database reflects a dual response involving cytotoxic effects and adaptive signaling which suggests suppression of cellular metabolism leading to reduced cancer cell growth and survival [53].

NF-κB plays crucial roles in cellular stress responses and inflammation [54]. The couple function between NF-κB and p53 might be simultaneously activated by DNA damage or cytokines resulting in the induction of cell death. This suggests that NF-κB contributes more to the execution phase of apoptosis rather than its initiation [55]. In addition, NF-κB was induced by DNA-damaging agents through receptor-independent mechanisms resulting in responses to diverse forms of genotoxic stress, including SN1-type DNA methylation induced by temozolomide, DNA crosslinking caused by cisplatin, and double-strand breaks generated by ionizing radiation [56]. Moreover, NF-κB expression was upregulated in HT-29 cells following treatment with chemotherapeutic agents such as fluorouracil, oxaliplatin, or paclitaxel [57]. Similarly, previous studies showed that adenosine induced G0/G1 cell cycle arrest in hepatocellular carcinoma cells, accompanied by increased caspase-3 activity and upregulation of p53. Concurrently, NF-κB expression was elevated, while the anti-apoptotic protein Bcl-2 was downregulated [58].

Activation of the MAPK and JAK/STAT pathways in response to sorafenib likely reflects compensatory survival signaling. The cancer cells could activate alternative pathways to counteract sorafenib effects. Particularly, inhibition of RAF kinases by sorafenib may paradoxically reactivate downstream MAPK/ERK signaling resulting in restoration of MAPK activity which occurs in a dose-dependent manner and contributes to continued cancer cell survival [59]. While the MAPK pathway generally drives cell proliferation and survival, the MAPK branches known as JNK and p38 were predominantly activated by environmental stressors, cytokines, and UV radiation to mediate apoptosis [60,61]. Similarly, activation of the JAK/STAT pathway supports cell survival under therapeutic pressure. These adaptive responses are further supported by the activation of additional signaling pathways, including PI3K/Akt and receptor tyrosine kinases, highlighting the dynamic rewiring of signaling networks that limits the efficacy of sorafenib in hepatocellular carcinoma [62,63]. Notably, a previous study reported that the short-chain fatty acid sodium propionate inhibited JAK2–STAT3 activation, induced cell cycle arrest, and promoted ROS generation, which in turn activated p38 and triggered apoptosis [64]. Notably, p53 and apoptosis pathways were also markedly upregulated following sorafenib treatment. These findings are consistent with our experimental observations demonstrating increased ROS generation and apoptosis. The concurrent upregulation of both MAPK and p53 signaling pathways in our study reflects a paradoxical feature of tumor biology, in which pro-survival (MAPK) and tumor-suppressive (p53) mechanisms were activated simultaneously. It is suggested that cancer cells experience high levels of intrinsic and treatment-induced stress such as DNA damage, ROS, and metabolic pressure, leading to the simultaneous engagement of both survival and stress-induced cell death pathways [65,66,67,68]. These findings assumed that sorafenib exerts anti-tumor effects through metabolic suppression and oxidative stress induction, while simultaneously triggering compensatory survival pathways that may limit its therapeutic efficacy.

Cannabis treatment alone induced a broad cytotoxic transcriptional response, characterized by activation of oxidative stress, inflammatory signaling, and apoptosis, along with suppression of proliferation, metabolism, and DNA repair pathways. Specifically, the upregulation of TNF/NF-κB, TGF-β, and IL-6/JAK–STAT signaling, together with enrichment of ROS and xenobiotic metabolism pathways, indicates that cannabis elicits substantial oxidative and inflammatory stress. This is further supported by activation of p53 signaling and apoptosis, reflecting engagement of programmed cell death mechanisms. These findings are consistent with previous reports showing that cannabinoids induce ROS production and cytokine-mediated signaling, ultimately leading to apoptosis [69]. The p53 and apoptosis pathways are well recognized as key regulators of cell cycle control and cell death in various cancer cells. Several plant-derived compounds, particularly THC and CBD, have been reported to modulate p53 signaling and apoptotic processes through generation of ROS. The ROS play dual roles: (1) as upstream signals that activate p53, and (2) as downstream mediators that promote apoptosis. Furthermore, p53 activation suppress the expression of antioxidant genes, leading to enhanced oxidative stress and ultimately resulting in cell death [70,71]. This is strongly supported by our experimental findings of increased ROS production and apoptosis in cannabis-treated cells. Consistently, the activation of p53 and apoptosis pathways observed in this study supports a ROS-driven cytotoxic mechanism.

The finding of downregulation in cell cycle regulators (G2/M checkpoint, E2F targets), DNA replication and repair pathways, and metabolic processes, including glycolysis and fatty acid metabolism, indicated the impairment of proliferative capacity and the reduction in cellular ability to recover from damage. Previous publications showed that CBD induced anti-tumor effects by causing localized inflammation, oxidative stress, and ER stress leading to apoptosis [72]. Several reports demonstrated that CBD treatment induced cell cycle disturbances, cellular apoptosis, and ER stress in HepG2 and gastric cancer cells [30,73]. Moreover, THC also affected the downregulation of cell cycle progression resulting in an inhibition of cell proliferation. Notably, THC suppressed DNA repair pathways, including mismatch repair and base excision repair, suggesting a reduced capacity for genomic maintenance under stress conditions [74]. In breast cancer cell lines, the activation of CB2 receptor by THC showed the suppression of cell proliferation via the inhibition of cell cycle and induction of apoptosis. Specifically, THC induced cell cycle arrest at the G2/M phase through downregulation of cell division control protein 2 (Cdc2), as evidenced by reduced sensitivity to THC in Cdc2-overexpressing cells [75]. In our study, we found the downregulation of glycolysis, fatty acid metabolism, and peroxisome-associated pathways indicated a global disruption of metabolic cellular function. In contrast, the concurrent activation of PI3K/Akt/mTOR signaling pathways likely represented a compensatory survival response to stress-induced damage, reflecting a dynamic balance between pro-death and pro-survival signaling [76]. A previous study showed that activation of mechanistic target of rapamycin complex 1 (mTORC1) was mediated by ER stress, which induced the inositol-requiring enzyme 1- c-Jun NH2-terminal kinases (IRE1-JNK) pathway and apoptosis. Activation of mTORC1 also reduced Akt phosphorylation, an upstream event in IRE1-JNK signaling that contributed to apoptosis [77]. Besides cell survival response, PI3K/Akt signaling directly and indirectly regulates glucose, lipid, nucleotide, and amino acid metabolism, thereby supporting tumor cell proliferation and survival [78]. The phosphorylation of IRS-1 was reduced by THC leading to promote the PI3K/Akt pathway associated with enhanced glucose uptake, suggesting a link between metabolic modulation and activation of pro-survival signaling pathways [79]. This interplay reflects a dynamic adaptation in which metabolic suppression coexists with activation of survival pathways, ultimately highlighting a balance between stress-induced cytotoxicity and compensatory signaling that may influence treatment response. In addition to PI3K/Akt-mediated metabolic regulation, p53 also serves as a regulator of lipid metabolism, nucleotide biosynthesis, redox homeostasis, and energy production. Specifically, p53 suppressed glycolysis while promoting oxidative phosphorylation through transcriptional control of metabolic genes contributing to the inhibition of tumorigenesis [80]. In the context of the present findings, activation of p53 could contribute to the observed suppression of glycolytic and metabolic pathways, reinforcing its role in mediating anti-proliferative and tumor-suppressive effects.

Consistent with our observation of metabolic gene downregulation, cannabis was reported to disrupt cancer cell metabolism by depleting metabolites and suppressing metabolic pathways, ultimately reducing cell viability and promoting cell death. Mechanistically, cannabinoids acting through CB1 and CB2 receptors impair glycolytic activity, as reflected by an increased adenosine monophosphate/adenosine triphosphate (AMP/ATP) ratio, indicating disruption of cellular energy homeostasis. In addition, cannabinoid-induced autophagy has been linked to early ROS generation, which exacerbates metabolic stress and further compromises energy metabolism, as demonstrated in pancreatic adenocarcinoma models [81,82]. Hence, our findings could propose that cannabis exerted a multi-layered cytotoxic effect by inducing oxidative stress and apoptosis while simultaneously impairing proliferation, metabolism, and DNA repair capacity contributing to potential anticancer activity. The simultaneous induction of oxidative stress and suppression of DNA repair pathways may further exacerbate genomic instability, thereby sensitizing cells to apoptosis.

The combined treatment with sorafenib and cannabis triggered a broad and integrated stress response, involving inflammatory pathways and apoptotic pathways, including TNF/NF-κB, p53, IL2/IL-6-JAK-STAT, and TGF-β, as well as oxidative signaling pathways and ER-associated protein processing. Concurrent activation of PI3K/Akt/mTOR, wingless-related integration site/beta-catenin (Wnt/β-catenin), and Notch pathways likely reflect compensatory survival signaling in response to heightened cellular stress. In parallel, the suppression of proliferative and metabolic programs, as evidenced by downregulation of E2F-driven DNA replication, G2/M cell cycle progression, glycolysis, fatty acid metabolism, peroxisome function, and bile metabolism suggested a shift toward growth arrest and metabolic collapse. This coordinated change assumed that cannabis may improve the effect of sorafenib by blocking cancer cells’ survival mechanisms and increasing its ability to inhibit cell growth. Our findings are supported by previous experimental studies demonstrating that cannabinoid-based combination therapies enhanced cell cycle disruption and apoptosis in cancer cells. Co-treatment of THC, CBD, and cisplatin significantly increased G2/M phase arrest and elevated apoptotic cell populations in cervical cancer cells [83]. Furthermore, the combination of cabozantinib and CBD significantly enhanced apoptotic signaling by activation of stress and apoptosis-related regulators, including phosphorylated Chk2, c-Jun, and p53 [33]. Likewise, this is concordant with our transcriptomic data demonstrating activation of p53 signaling and apoptosis pathways in the combination group.

Although GSEA indicated enrichment of the epithelial–mesenchymal transition (EMT) gene set in the combination treatment, key features of a classical EMT program were not observed. Canonical EMT involves coordinated activation of transcription factors such as *SNAI1*/*2*, *ZEB1*/*2*, and *TWIST1*/*2*, together with upregulation of mesenchymal markers including *VIM*, *CDH2*, and *FN1* [84,85,86]. In our dataset, these markers showed negative ranking and were not part of the leading-edge subset, indicating limited contribution to the enrichment signal. Instead, the enrichment was primarily driven by genes associated with inflammatory and stress responses, such as *CXCL8*, *CXCL1*, *JUN*, and *GADD45A*/*B*, which are known to overlap with EMT-related signatures and can be induced by ROS [87,88] which was consistent with the finding of ROS elevation in the treatment group. Taken together, these findings suggest that the observed EMT enrichment reflects a partial and context-dependent transcriptional response rather than a coordinated EMT phenotype associated with increased invasiveness or metastatic potential.

## 4. Materials and Methods

### 4.1. Cannabis Extract

Cannabis extract was purchased from CM Phytotech Co., Ltd. (Chiang Mai, Thailand). The extract was prepared from cannabis inflorescences obtained from a cultivation farm in Mae Chaem District, Chiang Mai Province. The plant material was extracted using the Soxhlet extraction method. The extracts were subsequently submitted to the National Science and Technology Development Agency (NSTDA) (Pathum Thani, Thailand) for quantitative analysis of major cannabinoid compounds using an in-house method based on AOAC Official Method 2018.11 by high-performance liquid chromatography with limits of detection (LOD of CBD = 0.00014, LOD of CBDA = 0.00008% *w*/*w*, LOD of Δ^9^-THC = 0.00014% *w*/*w*, LOD of THCA = 0.00016% *w*/*w*).

### 4.2. Cell Culture

The human hepatocellular carcinoma (HepG2, ATCC HB-8065) cell line was kindly provided by Assoc. Prof. Dr. Pornsiri Pitchakarn, Department of Biochemistry, Faculty of Medicine, Chiang Mai University. The cell line was grown in Dulbecco’s Modified Eagle Medium (DMEM) (GibGO, Grand Island, NY, USA), supplemented with 10% fetal bovine serum, penicillin and streptomycin in monolayer culture flasks at 37 °C, 5% CO_2_ and 95% air atmosphere at constant humidity. The cells were subcultured every 2–3 days prior to the experiments. For the experiments, the cells were harvested, washed with phosphate-buffered saline (PBS), and centrifuged at 200× *g* for 2 min. Cell viability was determined using trypan blue staining, and the cell density was adjusted to the required concentration.

### 4.3. Cell Viability

The cell viability was evaluated using the 3-(4,5-dimethylthiazol-2-yl)-2,5-diphenyl tetrazolium bromide (MTT) assay as previously described by Acquavia et al., 2023 [89]. Briefly, HepG2 cells were seeded into 96-well microplates at a density of 5 × 10^4^ cell/well and incubated for 24 h to reach approximately 70–80% confluence. The cells were then treated with cannabis extract or sorafenib alone. Cell viability was then assessed using the MTT assay. After 24 h of exposure, the culture medium was removed and replaced with MTT solution (2 mg/mL) (Sigma-Aldrich, Burlington, MO, USA). Following incubation for 4 h at 37 °C in the dark, the resulting formazan crystals were dissolved in dimethyl sulfoxide (DMSO) (Sigma-Aldrich, Burlington, MO, USA). The absorbance was measured at 540 nm and 630 nm using a microplate reader (BioTek, Winooski, VT, USA). Cell viability was expressed as a percentage relative to the vehicle control. Briefly, the IC_50_ values were calculated using nonlinear regression analysis based on a four-parameter logistic (4PL) model implemented in GraphPad Prism version 10.2.3 for Windows, GraphPad Software, Boston, MA, USA, and sigmoidal dose–response curves were generated using an online platform (https://www.aatbio.com/tools/ic50-calculator, accessed on 26 April 2026).

### 4.4. Drug Interaction

Based on the IC_50_ values obtained from previous experiments, concentrations covering the IC_50_ were selected for drug interaction studies. HepG2 cells were treated with combinations of cannabis extract and sorafenib for 24 h. Cell viability was then assessed using the MTT assay. The procedure was followed by the protocol from the previous experiment. Cell viability was expressed as a percentage relative to the vehicle control. The combination index and synergistic interactions were calculated and analyzed using the Combenefit software version 2.021 [90]. Combenefit is a software tool that enables the visualization, analysis and quantification of substance combination effects. Data obtained from combination treatments were analyzed using three reference synergy models: the Highest Single Agent (HSA) model, the Loewe additivity model, and the Bliss independence model. To confirm the pattern of drug interaction, SynergyFinder, a web-based platform, was performed to analyze drug interaction [91]. Drug–drug interactions were evaluated using three reference models: HSA, Bliss independence, and Loewe additivity. The HSA model compares the observed combination effect to the maximal effect of either single agent, providing a conservative estimate of synergy. The Bliss model assumes that the two compounds act independently through different mechanisms, and calculates the expected combined effect based on probabilistic independence. In contrast, the Loewe model assumes that the two agents have similar or overlapping mechanisms of action and evaluates whether one drug can be substituted by the other. Synergy, additivity, or antagonism were determined by comparing observed responses to the expected effects under each model [22,23,24].

### 4.5. RNA Extraction

HepG2 cells were seeded into 12-well microplates at a density of 4 × 10^5^ cell/well and incubated for 24 h to reach approximately 70–80% confluence. After 24 h of incubation, the cells were treated with cannabis extract and/or sorafenib at significant concentrations from the Combenefit software for an additional 24 h. The cells were then harvested by washing twice with ice-cold phosphate-buffered saline (PBS). Total RNA was extracted using the RNAeasy Fast Tissue/Cell Kit (Tiangen, Beijing, China) according to the manufacturer’s instructions. After treatment, the cells were collected using trypsin–EDTA solution (GibGO, Grand Island, NY, USA), and lysis buffer was subsequently added. The lysed cells were centrifuged at 13,400× *g* for 5 min to obtain the supernatant. The supernatant was then transferred to a DNA Eraser column and centrifuged at 13,400× *g* for 30 s. Subsequently, 70% ethanol was added, and the mixture was transferred to an RNase-Free Column CR4 and centrifuged at 13,400× *g* for 30 s. Buffer RW, pre-mixed with absolute ethanol, was added and incubated at room temperature for 2 min, followed by centrifugation. Finally, RNA was eluted using RNase-free ddH_2_O. The concentration and purity of the extracted RNA were assessed using a NanoDrop spectrophotometer (Thermo Fisher Scientific, Waltham, MA, USA). RNA integrity was further evaluated by agarose gel electrophoresis as shown in Supplementary File S5.

### 4.6. Transcriptomic Analysis

The obtained RNA was shipped to NovogeneAIT Genomics Singapore Pte., Ltd. (Singapore) to perform RNA-seq. The quality of the obtained RNA was assessed using 5400 Fragment Analyzer (Agilent Technologies, Inc., Santa Clara, CA, USA) analysis and an RNA integrity number range from 9.70 to 9.80 for all samples. Messenger RNA was purified from total RNA using poly-T oligo-attached magnetic beads. After fragmentation, the first strand cDNA was synthesized using random hexamer primers (NovogeneAIT, Helios, Singapore). The library was checked with Qubit. The real-time PCR and bioanalyzer were used to quantify and detect distribution of size. After library quality control, different libraries were pooled based on the effective concentration and targeted data amount and then subjected to Illumina sequencing.

To quantify gene expression level, featureCounts (2.0.6) was used to count the read numbers mapped to each gene. Then, Fragments Per Kilobase of transcript per Million mapped reads (FPKM) of each gene was calculated based on the length of the gene and reads count mapped to this gene. Regarding differential expression analysis (DEG), this was performed using the DESeq2 R package (1.42.0). The resulting *p*-value is adjusted using the Benjamini and Hochberg method to control the error discovery rate. The threshold of significant differential expression at padj ≤ 0.05 and |log2(foldchange)| ≥ 1. Gene Ontology (GO) terms with a corrected *p*-value of less than 0.05 were considered significantly enriched by differentially expressed genes. GO enrichment analysis of differentially expressed genes was implemented by the clusterProfiler (4.8.1), in which gene 4 length bias was corrected. To investigate pathway-level alterations associated with differentially expressed genes (DEGs), Gene Set Enrichment Analysis (GSEA) was performed using GSEA software version 4.4.0 [92,93]. All genes were ranked based on differential expression between treatment and control groups using the log2 fold change and corresponding statistical significance derived from RNA-sequencing analysis. The h.all.v5.1.symbols.gmt [Hallmark] gene set collection from the Molecular Signatures Database (MSigDB) was used for enrichment analysis with 1000 permutations. Gene sets with a nominal *p*-value < 0.05 and a false discovery rate (FDR) q-value < 0.05 were considered significantly enriched. Kyoto Encyclopedia of Genes and Genomes (KEGG) is a database resource for understanding high-level functions and utilities of the biological system, such as the cell, the organism and the ecosystem, from molecular-level information, especially large-scale molecular datasets generated by genome sequencing and other high-throughput experimental technologies (http://www.genome.jp/kegg/, accessed on 24 April 2026). We used ShinyGO 0.85.1 to access the statistical enrichment of differentially expressed genes in KEGG pathways [94,95].

### 4.7. ROS Staining

HepG2 cells were seeded into 12-well microplates at a density of 4 × 10^5^ cell/well and incubated for 24 h to reach approximately 70–80% confluence. After 24 h of incubation, cells were treated with cannabis extract and/or sorafenib at the same concentration used in the transcriptomic experiment. Then, the cells were harvested using trypsin–EDTA solution. The cells were stained with 2′,7′-dichlorofluorescin diacetate (DCFDA) according to the manufacturer’s instructions (Sigma-Aldrich, Burlington, MO, USA). Two hundred microliters of 20 µM of 2′,7′-dichlorofluorescin diacetate was added. The cells were then incubated at 37 °C for 30 min. After incubation, the cells were washed twice with ice-cold phosphate-buffered saline (PBS). Finally, the fluorescent-stained cells were counted using a flow cytometer (Beckman Coulter, Pasadena, CA, USA). The result was expressed as the percentage of ROS-stained cells.

### 4.8. Apoptosis Analysis

HepG2 cells were seeded into 12-well microplates at a density of 4 × 10^5^ cell/well and incubated for 24 h to reach approximately 70–80% confluence. After 24 h of incubation, the cells were treated with cannabis extract and/or sorafenib at the same concentration used in the transcriptomic experiment. Then, the cells were harvested using trypsin–EDTA solution. The cells were stained with 5 µL of Annexin V-ABFluor^TM^ and 2 µL of propidium iodide for 15 min in the dark according to the manufacturer’s instructions (Abbkine, Wuhan, China). After incubation, the cells were resuspended with annexin binding buffer, and the fluorescent-stained cells were counted using a flow cytometer (Beckman Coulter, Pasadena, CA, USA). The result was expressed as the percentage of apoptotic cells.

### 4.9. Statistical Analysis

Statistical analyses were performed using GraphPad Prism version 10 (GraphPad Software, San Diego, CA, USA). Data are presented as the mean ± standard deviation (SD) from different independent experiments. Differences among groups were analyzed using one-way analysis of variance (ANOVA), followed by Dunnett’s post hoc test for multiple comparisons. A *p*-value of ≤0.05 was considered statistically significant.

## 5. Conclusions

The present findings provide initial evidence of a drug–drug interaction between the conventional anticancer agent sorafenib and cannabis extract. The study demonstrated that the combination of sorafenib and cannabis extract induced a distinct and enhanced transcriptional response compared with single treatments. Integrated RNA-sequencing, GSEA, ShinyGO, and GO analyses consistently revealed activation of stress- and inflammation-related pathways, together with p53-mediated apoptosis, alongside suppression of metabolic and proliferative processes. The combination further integrated oxidative and endoplasmic reticulum stress responses, indicating a multi-layered mechanism. These transcriptomic findings were supported by increased ROS production and apoptosis, with the strongest effects observed under the combination treatment. However, this study has several limitations, including the lack of validation using RT-qPCR or western blotting and the reliance on a single HCC cell line (HepG2). Therefore, validation in additional HCC cell lines with diverse genetic backgrounds, such as Huh-7, Hep3B, and HepaRG, will be essential to confirm the reproducibility and broader applicability of these findings. Furthermore, in vivo studies are required to establish the translational relevance of this combination strategy.

## Figures and Tables

**Table 1:** Top 20 differentially expressed genes (upregulated and downregulated) relative to vehicle control in HepG2 cells following treatment with sorafenib or cannabis extract or combined sorafenib and cannabis extract (n = 3).

	Genes		
	Sorafenib	Cannabis Extract	Combination
Upregulation	*MT-RNR2*, *ACTB*, *ACTG1*, *MT2A*, *MT1G*, *GDF15*, *SLC3A2*, *MT1E*, *EIF1*, *JAG1*, *NOTUM*, *ODC1*, *H3F3B*, *MT1H*, *MYC*, *TRIB3*, *PIM1*, *SQSTM1*, *GNA13*, *NFE2L2*	*FTL*, *ACTG1*, *MT2A*, *MT1G*, *GDF15*, *SLC3A2*, *MT1E*, *EIF1*, *IGFBP1*, *JAG1*, *TXNRD1*, *ERRFI1*, *DDX21*, *MT1H*, *MYC*, *GCLC*, *HSPA9*, *H1F0*, *SERPINE1*, *ATF4*	*MT-RNR2*, *MT2A*, *MT1G*, *MT-RNR1*, *GDF15*, *SLC3A2*, *MT1E*, *EIF1*, *JAG1*, *TXNRD1*, *MT1H*, *MYC*, *GCLC*, *TRIB3*, *SAT1*, *BRD2*, *PIM1*, *SQSTM1*, *GNA13*, *MTHFD2*
Downregulation	*SLC2A1*, *HSPA8*, *LDHA*, *IGFBP1*, *LYZ*, *SLC2A3*, *MAN1A1*, *PEG3*, *HNRNPA2B1*, *PGK1*, *RPS6KA3*, *SLC16A3*, *PGAM1*, *PLIN2*, *NORAD*, *TM4SF1*, *IDH1*, *BNIP3*, *ST6GAL1*, *TGFBR2*	*AFP*, *TF*, *PEG10*, *LDHA*, *FGG*, *SLC2A3*, *PEG3*, *PGK1*, *TPI1*, *FGA*, *APOH*, *FGB*, *P4HA1*, *HMGA2*, *SERPINF2*, *PLOD2*, *ANXA4*, *TNFRSF19*, *COL2A1*, *OBSL1*	*AFP*, *SLC2A1*, *SELENOP*, *LDHA*, *LYZ*, *SLC2A3*, *AGT*, *PEG3*, *FGA*, *RPS6KA3*, *PGAM1*, *IDH1*, *ST6GAL1*, *SERPIND1*, *CPLX2*, *MMD*, *HMGA2*, *MCM3*, *PLOD2*, *ENC1*

## Data Availability

The original contributions presented in this study are included in the article and Supplementary Materials. Further inquiries can be directed to the corresponding authors.

## Supplementary Materials

The following supporting information can be downloaded at https://www.mdpi.com/article/10.3390/ijms27104342/s1.

## References

[1] Bray F., Laversanne M., Sung H., Ferlay J., Siegel R.L., Soerjomataram I., Jemal A. (2024). Global cancer statistics 2022: GLOBOCAN estimates of incidence and mortality worldwide for 36 cancers in 185 countries. CA Cancer J. Clin..

[2] Nguyen D.T., Nguyen D.H., Nguyen V.T.H. (2023). Sorafenib as first-line treatment for patients with primary hepatocellular carcinoma: An outcome evaluation. J. Int. Med. Res..

[3] Zhai B., Sun X.Y. (2013). Mechanisms of resistance to sorafenib and the corresponding strategies in hepatocellular carcinoma. World J. Hepatol..

[4] Tang W., Chen Z., Zhang W., Cheng Y., Zhang B., Wu F., Wang Q., Wang S., Rong D., Reiter F.P. (2020). The mechanisms of sorafenib resistance in hepatocellular carcinoma: Theoretical basis and therapeutic aspects. Signal Transduct. Target. Ther..

[5] Ransing R., de la Rosa P.A., Pereira-Sanchez V., Handuleh J.I.M., Jerotic S., Gupta A.K., Karaliuniene R., de Filippis R., Peyron E., Sönmez Güngör E. (2022). Current state of cannabis use, policies, and research across sixteen countries: Cross-country comparisons and international perspectives. Trends Psychiatry Psychother..

[6] Esmaeli M., Dehghanpour Dehabadi M. (2025). Cannabidiol (CBD) as a potential therapeutic agent for liver cancer: A comprehensive review of current evidence. Cancer Cell Int..

[7] Graczyk M., Lewandowska A.A., Dzierżanowski T. (2021). The Therapeutic Potential of Cannabis in Counteracting Oxidative Stress and Inflammation. Molecules.

[8] Hanganu B., Lazar D.E., Manoilescu I.S., Mocanu V., Butcovan D., Buhas C.L., Szalontay A.S., Ioan B.G. (2022). Controversial Link between Cannabis and Anticancer Treatments-Where Are We and Where Are We Going? A Systematic Review of the Literature. Cancers.

[9] Whynot E.G., Tomko A.M., Dupré D.J. (2023). Anticancer properties of cannabidiol and Δ^9^-tetrahydrocannabinol and synergistic effects with gemcitabine and cisplatin in bladder cancer cell lines. J. Cannabis Res..

[10] Brunetti P., Pichini S., Pacifici R., Busardò F.P., Del Rio A. (2020). Herbal Preparations of Medical Cannabis: A Vademecum for Prescribing Doctors. Medicina.

[11] Aare M., Lazarte J.M., Nathani A., Boirie B., Turley T.N., Copland J.A., Singh M. (2026). Synergistic Anticancer Activity of Cannabinoids and Terpenes Against Triple-Negative Breast Cancer Resistance. Int. J. Mol. Sci..

[12] Marzęda P., Wróblewska-Łuczka P., Drozd M., Florek-Łuszczki M., Załuska-Ogryzek K., Łuszczki J.J. (2022). Cannabidiol Interacts Antagonistically with Cisplatin and Additively with Mitoxantrone in Various Melanoma Cell Lines—An Isobolographic Analysis. Int. J. Mol. Sci..

[13] Buchtova T., Beresova L., Chroma K., Pluhacek T., Beres T., Kaczorova D., Tarkowski P., Bartek J., Mistrik M. (2023). Cannabis-derived products antagonize platinum drugs by altered cellular transport. Biomed. Pharmacother..

[14] Bar-Sela G., Cohen I., Campisi-Pinto S., Lewitus G.M., Oz-Ari L., Jehassi A., Peer A., Turgeman I., Vernicova O., Berman P. (2020). Cannabis Consumption Used by Cancer Patients during Immunotherapy Correlates with Poor Clinical Outcome. Cancers.

[15] Afrin F., Chi M., Eamens A.L., Duchatel R.J., Douglas A.M., Schneider J., Gedye C., Woldu A.S., Dun M.D. (2020). Can Hemp Help? Low-THC Cannabis and Non-THC Cannabinoids for the Treatment of Cancer. Cancers.

[16] Woerdenbag H.J., Olinga P., Kok E.A., Brugman D.A.P., van Ark U.F., Ramcharan A.S., Lebbink P.W., Hoogwater F.J.H., Knapen D.G., de Groot D.J.A. (2023). Potential, Limitations and Risks of Cannabis-Derived Products in Cancer Treatment. Cancers.

[17] Suttithumsatid W., Sukketsiri W., Panichayupakaranant P. (2023). Cannabinoids and standardized cannabis extracts inhibit migration, invasion, and induce apoptosis in MCF-7 cells through FAK/MAPK/Akt/NF-κB signaling. Toxicol. Vitr..

[18] Schoeman R., de la Harpe A., Beukes N., Frost C.L. (2022). Cannabis with breast cancer treatment: Propitious or pernicious?. 3 Biotech..

[19] Śledziński P., Zeyland J., Słomski R., Nowak A. (2018). The current state and future perspectives of cannabinoids in cancer biology. Cancer Med..

[20] Takeda S., Yamamoto I., Watanabe K. (2009). Modulation of Δ^9^-tetrahydrocannabinol-induced MCF-7 breast cancer cell growth by cyclooxygenase and aromatase. Toxicology.

[21] Blasco-Benito S., Seijo-Vila M., Caro-Villalobos M., Tundidor I., Andradas C., García-Taboada E., Wade J., Smith S., Guzmán M., Pérez-Gómez E. (2018). Appraising the “entourage effect”: Antitumor action of a pure cannabinoid versus a botanical drug preparation in preclinical models of breast cancer. Biochem. Pharmacol..

[22] Mao B., Guo S. (2023). Statistical Assessment of Drug Synergy from In Vivo Combination Studies Using Mouse Tumor Models. Cancer Res. Commun..

[23] Kashif M., Andersson C., Mansoori S., Larsson R., Nygren P., Gustafsson M.G. (2017). Bliss and Loewe interaction analyses of clinically relevant drug combinations in human colon cancer cell lines reveal complex patterns of synergy and antagonism. Oncotarget.

[24] Lederer S., Dijkstra T.M.H., Heskes T. (2018). Additive Dose Response Models: Explicit Formulation and the Loewe Additivity Consistency Condition. Front. Pharmacol..

[25] Gauthier A., Ho M. (2013). Role of sorafenib in the treatment of advanced hepatocellular carcinoma: An update. Hepatol. Res..

[26] Faiz M.B., Naeem F., Irfan M., Aslam M.A., Estevinho L.M., Ateşşahin D.A., Alshahrani A.M., Calina D., Khan K., Sharifi-Rad J. (2024). Exploring the therapeutic potential of cannabinoids in cancer by modulating signaling pathways and addressing clinical challenges. Discov. Oncol..

[27] Ye Q., Gui C., Jin D., Zhang J., Zhang J., Ma N., Xu L. (2024). Synergistic effect of cannabidiol with dasatinib on lung cancer by SRC/PI3K/AKT signal pathway. Biomed. Pharmacother..

[28] Lamtha T., Jongkon N., Lertvanithphol T., Horprathum M., Seetaha S., Choowongkomon K. (2025). Cannabinoids as Promising Inhibitors of HER2-Tyrosine Kinase: A Novel Strategy for Targeting HER2-Positive Ovarian Cancer. ACS Omega.

[29] Deng L., Ng L., Ozawa T., Stella N. (2017). Quantitative Analyses of Synergistic Responses between Cannabidiol and DNA-Damaging Agents on the Proliferation and Viability of Glioblastoma and Neural Progenitor Cells in Culture. J. Pharmacol. Exp. Ther..

[30] Chen S., Li X., Wu Q., Li Y., Puig M., Moulin F., Choudhuri S., Gingrich J., Guo L. (2024). Investigation of cannabidiol-induced cytotoxicity in human hepatic cells. Toxicology.

[31] Di Giacomo S., Briz O., Monte M.J., Sanchez-Vicente L., Abete L., Lozano E., Mazzanti G., Di Sotto A., Marin J.J.G. (2019). Chemosensitization of hepatocellular carcinoma cells to sorafenib by β-caryophyllene oxide-induced inhibition of ABC export pumps. Arch. Toxicol..

[32] Go Y.Y., Kim S.R., Kim D.Y., Chae S.W., Song J.J. (2020). Cannabidiol enhances cytotoxicity of anti-cancer drugs in human head and neck squamous cell carcinoma. Sci. Rep..

[33] Jeon Y., Kim T., Kwon H., Kim J.-K., Park Y.-T., Ham J., Kim Y.-J. (2023). Cannabidiol Enhances Cabozantinib-Induced Apoptotic Cell Death via Phosphorylation of p53 Regulated by ER Stress in Hepatocellular Carcinoma. Cancers.

[34] Melones-Herrero J., Delgado-Aliseda P., Figueiras S., Velázquez-Gutiérrez J., Quiroga A.G., Calés C., Sánchez-Pérez I. (2024). Trans-[Pt(amine)Cl_2_(PPh_3_)] Complexes Target Mitochondria and Endoplasmic Reticulum in Gastric Cancer Cells. Int. J. Mol. Sci..

[35] Ye L., Yang X., Guo E., Chen W., Lu L., Wang Y., Peng X., Yan T., Zhou F., Liu Z. (2014). Sorafenib metabolism is significantly altered in the liver tumor tissue of hepatocellular carcinoma patient. PLoS ONE.

[36] Chayasirisobhon S. (2020). Mechanisms of Action and Pharmacokinetics of Cannabis. Perm. J..

[37] Pegg A.E. (2008). Spermidine/spermine-N1-acetyltransferase: A key metabolic regulator. Am. J. Physiol.-Endocrinol. Metab..

[38] Wang C., Ruan P., Zhao Y., Li X., Wang J., Wu X., Liu T., Wang S., Hou J., Li W. (2017). Spermidine/spermine N1-acetyltransferase regulates cell growth and metastasis via AKT/β-catenin signaling pathways in hepatocellular and colorectal carcinoma cells. Oncotarget.

[39] Qi M., Zhou Y., Liu J., Ou X., Li M., Long X., Ye J., Yu G. (2018). AngII induces HepG2 cells to activate epithelial-mesenchymal transition. Exp. Ther. Med..

[40] Tang W.-G., Feng J.-F., Li X., Sun Q.-M., Hu J.-W., Ma X.-L., Nie Y.-Y., Xu Y., Sun J., Chang Q.-M. (2025). MCM3 promotes hepatocellular carcinoma progression via Epithelial-mesenchymal Transition through AKT/Twist signaling pathway. Ann. Hepatol..

[41] Li K., Niu Y., Li K., Zhong C., Qiu Z., Yuan Y., Shi Y., Lin Z., Huang Z., Zuo D. (2023). Dysregulation of PLOD2 Promotes Tumor Metastasis and Invasion in Hepatocellular Carcinoma. J. Clin. Transl. Hepatol..

[42] Liu H., Liu D., Li Z. (2023). Expression and clinical significance of ENC1 in gastrointestinal tumors: Bioinformatics analysis based on a public gene database. J. Gastrointest. Oncol..

[43] Zhou Y., Tang X., Niu L., Liu Y., Wang B., He J. (2020). Ectodermal-neural cortex 1 as a novel biomarker predicts poor prognosis and induces metastasis in breast cancer by promoting Wnt/β-catenin pathway. J. Cell Mol. Med..

[44] Licari E., Sánchez-del-Campo L., Falletta P. (2021). The two faces of the Integrated Stress Response in cancer progression and therapeutic strategies. Int. J. Biochem. Cell Biol..

[45] Román-Trufero M., Kleijn I.T., Blighe K., Zhou J., Saavedra-García P., Gaffar A., Christoforou M., Bellotti A., Abrahams J., Atrih A. (2025). An ISR-independent role of GCN2 prevents excessive ribosome biogenesis and mRNA translation. Life Sci. Alliance.

[46] Waldmann T.A. (2018). Cytokines in Cancer Immunotherapy. Cold Spring Harb. Perspect. Biol..

[47] Gong F., Miller K.M. (2013). Mammalian DNA repair: HATs and HDACs make their mark through histone acetylation. Mutat. Res..

[48] Lin R., Zhang Y., Li H., Liang F. (2026). The role of histone acetyltransferases in tumorigenesis and their therapeutic potential: A review. Biochem. Biophys. Res. Commun..

[49] Yang X., Bam M., Nagarkatti P.S., Nagarkatti M. (2019). Cannabidiol Regulates Gene Expression in Encephalitogenic T cells Using Histone Methylation and noncoding RNA during Experimental Autoimmune Encephalomyelitis. Sci. Rep..

[50] Yang X., Hegde V.L., Rao R., Zhang J., Nagarkatti P.S., Nagarkatti M. (2014). Histone modifications are associated with Δ^9^-tetrahydrocannabinol-mediated alterations in antigen-specific T cell responses. J. Biol. Chem..

[51] Prini P., Penna F., Sciuccati E., Alberio T., Rubino T. (2017). Chronic Δ^8^-THC Exposure Differently Affects Histone Modifications in the Adolescent and Adult Rat Brain. Int. J. Mol. Sci..

[52] Izumi M., Fujii M., Kobayashi I.S., Ho V., Kashima Y., Udagawa H., Costa D.B., Kobayashi S.S. (2024). Integrative single-cell RNA-seq and spatial transcriptomics analyses reveal diverse apoptosis-related gene expression profiles in EGFR-mutated lung cancer. Cell Death Dis..

[53] Yu M., Yang D., Chen X., Yang Y., Zhang B., Jiang X., Xing L., Yang Y., Sun Y., Li N. (2026). Metabolic reprogramming in cancer: Dysregulation of glucose, lipid, and amino acid pathways and therapeutic opportunities. Mol. Biomed..

[54] Chichirau B.E., Diechler S., Posselt G., Wessler S. (2019). Tyrosine Kinases in Helicobacter pylori Infections and Gastric Cancer. Toxins.

[55] Ryan K.M., Ernst M.K., Rice N.R., Vousden K.H. (2000). Role of NF-kappaB in p53-mediated programmed cell death. Nature.

[56] Wang W., Mani A.M., Wu Z.H. (2017). DNA damage-induced nuclear factor-kappa B activation and its roles in cancer progression. J. Cancer Metastasis Treat..

[57] Liu T., Liu D., Liu J., Song J.T., Gao S.L., Li H., Hu L.H., Liu B.R. (2012). Effect of NF-κB inhibitors on the chemotherapy-induced apoptosis of the colon cancer cell line HT-29. Exp. Ther. Med..

[58] Wu L.-F., Li G.-P., Su J.-D., Pu Z., Feng J.-L., Ye Y.-Q., Wei B. (2010). Involvement of NF-kappaB activation in the apoptosis induced by extracellular adenosine in human hepatocellular carcinoma HepG2 cells. Biochem. Cell Biol..

[59] Chen Y., Liu Y.-C., Sung Y.-C., Ramjiawan R.R., Lin T.-T., Chang C.-C., Jeng K.-S., Chang C.-F., Liu C.-H., Gao D.-Y. (2017). Overcoming sorafenib evasion in hepatocellular carcinoma using CXCR4-targeted nanoparticles to co-deliver MEK-inhibitors. Sci. Rep..

[60] Song Y., Du Y., Li W., Fan Y., Zhang Y., Shen H., Cheng L., Wang J., Deng F., Tang B. (2025). Targeting the p38/MAPK pathway to induce apoptosis: A multidimensional mechanistic exploration of Mentha and its active compound diosmetin against liver cancer. Sci. Rep..

[61] Wang X., Ron D. (1996). Stress-Induced Phosphorylation and Activation of the Transcription Factor CHOP (GADD153) by p38 MAP Kinase. Science.

[62] Zhu Y.J., Zheng B., Wang H.Y., Chen L. (2017). New knowledge of the mechanisms of sorafenib resistance in liver cancer. Acta Pharmacol. Sin..

[63] Vij P., Hussain M.S., Satapathy S.K., Cobos E., Tripathi M.K. (2024). The Emerging Role of Long Noncoding RNAs in Sorafenib Resistance Within Hepatocellular Carcinoma. Cancers.

[64] Park H.-S., Han J.-H., Park J.W., Lee D.-H., Jang K.-W., Lee M., Heo K.-S., Myung C.-S. (2021). Sodium propionate exerts anticancer effect in mice bearing breast cancer cell xenograft by regulating JAK2/STAT3/ROS/p38 MAPK signaling. Acta Pharmacol. Sin..

[65] Hanson R.L., Batchelor E. (2022). Coordination of MAPK and p53 dynamics in the cellular responses to DNA damage and oxidative stress. Mol. Syst. Biol..

[66] Wu Y., Zhou B.P. (2010). TNF-α/NF-κB/Snail pathway in cancer cell migration and invasion. Br. J. Cancer.

[67] Stramucci L., Pranteda A., Bossi G. (2018). Insights of Crosstalk between p53 Protein and the MKK3/MKK6/p38 MAPK Signaling Pathway in Cancer. Cancers.

[68] Melones-Herrero J., Alcalá S., Ruiz-Cañas L., Benítez-Buelga C., Batres-Ramos S., Calés C., Lorenzo O., Perona R., Quiroga A.G., Sainz B. (2024). Platinum iodido drugs show potential anti-tumor activity, affecting cancer cell metabolism and inducing ROS and senescence in gastrointestinal cancer cells. Commun. Biol..

[69] Morales P., Hurst D.P., Reggio P.H. (2017). Molecular Targets of the Phytocannabinoids: A Complex Picture. Prog. Chem. Org. Nat. Prod..

[70] Wang F., Dezfouli A.B., Khosravi M., Sievert W., Stangl S., Schwab M., Wu Z., Steiger K., Ma H., Multhoff G. (2023). Cannabidiol-induced crosstalk of apoptosis and macroautophagy in colorectal cancer cells involves p53 and Hsp70. Cell Death Discov..

[71] Drane P., Bravard A., Bouvard V., May E. (2001). Reciprocal down-regulation of p53 and SOD2 gene expression-implication in p53 mediated apoptosis. Oncogene.

[72] Mokoena D., George B.P., Abrahamse H., Chakraborti S. (2022). The Role of Cannabis Species on Oxidative Stress in Cancer Cells. Handbook of Oxidative Stress in Cancer: Therapeutic Aspects.

[73] Zhang X., Qin Y., Pan Z., Li M., Liu X., Chen X., Qu G., Zhou L., Xu M., Zheng Q. (2019). Cannabidiol Induces Cell Cycle Arrest and Cell Apoptosis in Human Gastric Cancer SGC-7901 Cells. Biomolecules.

[74] Galanti G., Fisher T., Kventsel I., Shoham J., Gallily R., Mechoulam R., Lavie G., Amariglio N., Rechavi G., Toren A. (2008). Δ9-tetrahydrocannabinol inhibits cell cycle progression by downregulation of E2F1 in human glioblastoma multiforme cells. Acta Oncol..

[75] Caffarel M.M., Sarrió D., Palacios J., Guzmán M., Sánchez C. (2006). Δ9-tetrahydrocannabinol inhibits cell cycle progression in human breast cancer cells through Cdc2 regulation. Cancer Res..

[76] Pungsrinont T., Kallenbach J., Baniahmad A. (2021). Role of PI3K-AKT-mTOR Pathway as a Pro-Survival Signaling and Resistance-Mediating Mechanism to Therapy of Prostate Cancer. Int. J. Mol. Sci..

[77] Kato H., Nakajima S., Saito Y., Takahashi S., Katoh R., Kitamura M. (2012). mTORC1 serves ER stress-triggered apoptosis via selective activation of the IRE1–JNK pathway. Cell Death Differ..

[78] Su W.Y., Tian L.Y., Guo L.P., Huang L.Q., Gao W.Y. (2023). PI3K signaling-regulated metabolic reprogramming: From mechanism to application. Biochim. Biophys. Acta Rev. Cancer.

[79] Chen J.W., Kong Z.L., Tsai M.L., Lo C.Y., Ho C.T., Lai C.S. (2018). Tetrahydrocurcumin ameliorates free fatty acid-induced hepatic steatosis and improves insulin resistance in HepG2 cells. J. Food Drug Anal..

[80] Puzio-Kuter A.M. (2011). The Role of p53 in Metabolic Regulation. Genes. Cancer.

[81] Erukainure O.L., Oyenihi O.R., Amaku J.F., Chukwuma C.I., Nde A.L., Salau V.F., Matsabisa M.G. (2023). *Cannabis sativa* L. modulates altered metabolic pathways involved in key metabolisms in human breast cancer (MCF-7) cells: A metabolomics study. Heliyon.

[82] Dando I., Donadelli M., Costanzo C., Dalla Pozza E., D’Alessandro A., Zolla L., Palmieri M. (2013). Cannabinoids inhibit energetic metabolism and induce AMPK-dependent autophagy in pancreatic cancer cells. Cell Death Dis..

[83] Mathibela S.P., Lebelo M.T., Steenkamp V. (2026). Antiproliferative Effects of Cannabinoids and Cisplatin in Cervical Cancer Cells. Cancer Rep..

[84] Chattopadhyay I., Ambati R., Gundamaraju R. (2021). Exploring the Crosstalk between Inflammation and Epithelial-Mesenchymal Transition in Cancer. Mediat. Inflamm..

[85] Zeisberg M., Neilson E.G. (2009). Biomarkers for epithelial-mesenchymal transitions. J. Clin. Investig..

[86] Taube J.H., Herschkowitz J.I., Komurov K., Zhou A.Y., Gupta S., Yang J., Hartwell K., Onder T.T., Gupta P.B., Evans K.W. (2010). Core epithelial-to-mesenchymal transition interactome gene-expression signature is associated with claudin-low and metaplastic breast cancer subtypes. Proc. Natl. Acad. Sci. USA.

[87] Ha H., Debnath B., Neamati N. (2017). Role of the CXCL8-CXCR1/2 Axis in Cancer and Inflammatory Diseases. Theranostics.

[88] Liebermann D.A., Hoffman B. (2008). Gadd45 in stress signaling. J. Mol. Signal.

[89] Acquavia M.A., Tesoro C., Pascale R., Ostuni A., Matera I., Bianco G., Scrano L., Bufo S.A., Ciriello R., Di Capua A. (2023). Legal Cannabis sativa L. Dried Inflorescences: Cannabinoids Content and Cytotoxic Activity against Human HepG2 Cell Line. Appl. Sci..

[90] Di Veroli G.Y., Fornari C., Wang D., Mollard S., Bramhall J.L., Richards F.M., Jodrell D.I. (2016). Combenefit: An interactive platform for the analysis and visualization of drug combinations. Bioinformatics.

[91] Zheng S., Wang W., Aldahdooh J., Malyutina A., Shadbahr T., Tanoli Z., Pessia A., Tang J. (2022). SynergyFinder Plus: Toward Better Interpretation and Annotation of Drug Combination Screening Datasets. Genom. Proteom. Bioinform..

[92] Mootha V.K., Lindgren C.M., Eriksson K.F., Subramanian A., Sihag S., Lehar J., Puigserver P., Carlsson E., Ridderstråle M., Laurila E. (2003). PGC-1α-responsive genes involved in oxidative phosphorylation are coordinately downregulated in human diabetes. Nat. Genet..

[93] Subramanian A., Tamayo P., Mootha V.K., Mukherjee S., Ebert B.L., Gillette M.A., Paulovich A., Pomeroy S.L., Golub T.R., Lander E.S. (2005). Gene set enrichment analysis: A knowledge-based approach for interpreting genome-wide expression profiles. Proc. Natl. Acad. Sci. USA.

[94] Ge S.X., Jung D., Yao R. (2019). ShinyGO: A graphical gene-set enrichment tool for animals and plants. Bioinformatics.

[95] Luo W., Brouwer C. (2013). Pathview: An R/Bioconductor package for pathway-based data integration and visualization. Bioinformatics.

